# Prey Preference of the Predatory Mite, *Amblyseius swirskii* between First Instar Western Flower Thrips *Frankliniella occidentalis* and Nymphs of the Twospotted Spider Mite *Tetranychus urticae*

**DOI:** 10.1673/031.010.14109

**Published:** 2010-09-10

**Authors:** Xuenong Xu, Annie Enkegaard

**Affiliations:** ^1^ Key Laboratory for biological Control of Ministry of Agiculture, Institute of Plant Proctection, Chinese Academy of Agricultural Sciences, 2 West Yuanmingyuan Road, Beijing, 100193, P.R. Chinaction, Chinese Academy of China; ^2^ Department of Integrated Pest Management, Research Centre Flakkebjerg, Faculty of Agricultural Sciences, Aarhus University, Forsøgsvej 1, DK-4200 Slagelse, Denmark

**Keywords:** polyphagous, biological control

## Abstract

The prey preference of polyphagous predators plays an important role in suppressing different species of pest insects. In this study the prey preference of the predatory mite, *Amblyseius swirskii* (Athias-Henriot) (Acari: Phytoseiidae) was examined between nymphs of the twospotted spider mite, *Tetranychus urticae* Koch (Acari: Tetranychidae) and first instar larvae of the western flower thrips, *Frankliniella occidentalis* (Pergande) (Thysanoptera: Thripidae), as well as between active and chrysalis spider mite protonymphs and active and chrysalis spider mite deutonymphs. The study was done in the laboratory on bean leaf discs at 25 ± 1° C and 70 ± 5% RH. *Amblyseius swirskii* had a clear preference for thrips compared to both spider mite protonymphs and deutonymphs. About twice as many thrips as spider mites were consumed. *Amblyseius swirskii* did not show a preference between active and chrysalis stages of spider mites.

## Introduction

The western flower thrips *Frankliniella occidentalis* (Pergande) (Thysanoptera: Thripidae) and the twospotted spider mite *Tetranychus urticae* Koch (Acari: Tetranychidae) are economically important pests in many ornamentals and vegetables grown in greenhouses and fields all over the world (CAB International 2007; Helle and Sabelis 1985; Lewis 1997). The small size of the pests, their cryptic life form, fast reproduction, and ability to easily build up resistance to chemicals make them difficult to control with pesticides. Biological control of both pest species with various beneficials, especially predatory mites and pirate bugs, has therefore been practiced for a number of years, particularly in greenhouse crops (e.g. Bosco et al. 2008; Helle and Sabelis 1985; Jacobson et al. 2001; Tommasini and Maini 2001). Successful biocontrol can be obtained in many cases (e.g. Brødsgaard and Enkegaard 1997; Messelink et al. 2005, 2006) but especially biological control of thrips can be difficult in some cases, for instance in some ornamental crops (Parrella and Murphy 1996). Consequently the search continues for additional beneficials to be added to the existing assortment of commercially available beneficials against both pests (e.g. Sengonca et al. 2004; van der Linden 2004; Escudero and Ferragut 2005; van Houten et al. 2007b).

*Amblyseius swirskii* (Athias-Henriot) (Acari: Phytoseiidae) is a recent addition to the beneficial assortment, developed and marketed by the Dutch producer of beneficials, Koppert B.V. (van Houten et al. 2005). This mite is a polyphagous predator capable of preying on a number of food items, including gall mites (El-Laithy 1998), spider mites (Swirski et al. 1967; El-Laithy and Fouly 1992; Momen and El-Saway 1993; van Houten et al. 2007a), tarsonemid mites (Tal et al. 2007), citrus rust mites (Argov et al. 2007), whiteflies (Swirski et al. 1967; Nomikou et al. 2001, 2002; Hoogerbrugge et al. 2005; Calvo et al. 2006), thrips (van Houten et al. 2005; Messelink et al. 2005, 2006; Wimmer et al. 2008) and pollen (Ragusa and Swirski 1975; Nomikou et al. 2003). *Amblyseius swirskii* was marketed primarily for biocontrol of whiteflies and thrips, which however often occurs alongside other pest species in many greenhouse vegetables and ornamentals (Trichilo and Leigh 1986; Wilson et al. 1996; Pallini et al. 1998; Fejt and Jarosík 2000; Messelink and Janssen 2008; Trottin-Caudal et al. 2008).

Various aspects of the biology of *A. swirskii* have been examined, including fecundity (Ragusa and Swirski 1977; El-Laithy and Fouly 1992; Nomikou et al. 2001; Wimmer et al. 2008), predation capacity (Momen and El-Saway 1993; Tal et al. 2007) and temperature requirements (El-Tawab et al. 1982). In addition the prey preference of *A. swirskii* between whitefly and western flower thrips has been investigated (van Maanen and Janssen 2008) showing that it had some preference for first instar thrips larvae compared to whitefly eggs.

A number of the species in the prey range of *A. swirskii* are likely to coexist in crops, especially greenhouse ornamentals, subjected to biocontrol based on this predatory mite. Thus, although *A. swirskii* on its own does not cause satisfactory control of spider mites (van Houten et al. 2007a; Hoogerbrugge et al. 2009), its ability to prey on this pest may reduce the predation pressure on the target pest species intended to be controlled, e.g. thrips. Additional information about the prey preference of *A. swirskii* will therefore be valuable for evaluation of its control efficiency of target pests in multiple-pest environments. In this initial and basic laboratory study the prey preference of *A. swirskii* between spider mites and western flower thrips was examined. In addition the prey preference of *A. swirskii* between active spider mite nymphs and chrysalis forms was examined to evaluate if such a preference could influence the prey preference of *A. swirskii* between spider mites and thrips. Female predatory mites were chosen as the most voracious stage (Janssen et al. 2002); first instar thrips larvae were chosen since the predatory mite does not prey upon adult thrips and seldom on second larval instar (Wimmer et al. 2008); and spider mite nymphs were chosen due to their similarity in size and weight to thrips first instars (Zhang 2003; X. Xu unpublished data).

## Materials and methods

### Plant material

Bean seedlings (*Phaseolus vulgaris* var. Processor) were produced in thrips-proof net-covered cages (68 cm × 75 cm × 82 cm) in a greenhouse compartment at 25 ± 1° C, 70% ± 5% RH and 16:8 L:D at Research Centre Flakkebjerg, Faculty of Agricultural Sciences, Aarhus University, Denmark. The experiments were conducted on bean leaf discs cut from the first leaves of 2-week-old bean plants.

### Insects and mites

Western flower thrips and twospotted spider mites were reared separately on bean in similar cages and at similar conditions as above. For the experiment on prey preference, newly-emerged protonymphs or deutonymphs of spider mites and young 1^st^ instar larvae of thrips were used. Newly-emerged protonymphs and deutonymphs, which are slim and in general white or light-green in color, were harvested directly from the laboratory reared population . To standardize the age of the thrips larvae, cohorts of eggs were established by introducing fresh plants into the rearing cages for a two-day period followed by removal of adult thrips and subsequent transfer of the plants to separate cages. After another 2 days, young 1^st^ instar larvae (white to light green, slim, about 0.5– 0.7 mm length) could be harvested.

A stock colony of *A. swirskii* was initiated from a shipment obtained from Koppert B.V., the Netherlands, and reared in a climate cabinet at 25 ± 1° C, 70% ± 5% RH and 16:8 L:D in rearing set-ups with eggs of *Ephestia kuehniella* Zeller (Lepidoptera: Pyralidae) supplied twice a week as food. A rearing setup consisted of a Petri dish (diameter: 14 cm) with a sponge (diameter: 12 cm, height: 1 cm) placed on the bottom. A filter paper (diameter: 12 cm) and a piece of black plastic film (diameter: 11 cm) were positioned on the top of the sponge and predatory mites and prey were added to the black plastic film. The Petri dish was half filled with water to isolate the rearing arena. Prior to experimentation neonate adult females [maximum 2 days old; distinguished from older mites by their appearance (white, slim, even dorsal shield)] of *A. swirskii* were taken from the rearing to be individually transferred to a smaller rearing arenas (diameter: 2 cm) for starvation for 24 h.

### Prey preference between thrips and spider mites

The experimental set-up consisted of a bean leaf disc (diameter: 5.3 cm) placed upside down on a water-saturated cotton disc (diameter: 5.8 cm) in a Petri dish half filled with water.

Fifteen first instar larvae of western flower thrips and 15 protonymphs or deutonymphs of spider mites in the active stage were randomly chosen from the rearings and carefully transferred onto the experimental setup using a fine brush for 1 hour of acclimatization, after which one starved *A. swirskii* adult female was added. The set-ups were placed in a climatic chamber at 25 ± 1° C, 70% ± 5% RH and 16:8 L:D for 24 hours after which the number of consumed prey recorded. The experiment involving spider mite protonymphs was replicated 24 times with 3 controls (i.e. without predators), and the one involving spider mite deutonymphs was replicated 21 times with 4 controls, i.e. without predators. The number of replicates for the control treatment was determined on the basis of preliminary experiments in which no control mortality occurred in several replicates among thrips or among spider mites using the experimental procedure outlined above. Similarly, no mortality occurred in the controls during the experiments.

In the course of the experiments it was observed that some active spider mite nymphs switched to the chrysalis phase. Since this might influence the prey preference of *A. swirskii* between thrips and spider mites, the preference of the predatory mite between the active and chrysalis spider mite nymphs was examined.

### Prey preference between active and chrysalis stages of spider mite nymphs

The experimental set-up and conditions were as described above except that the experiment lasted only 12 h. Ten spider mites of both active and chrysalis stages of either proto- or deutonymphs were randomly put onto the bean leaf disc. The spider mite nymphs had been in the required stage for less than 3 h, hereby ensuring that the minimum time period (16 h; X. Xu perssonal observations) between transitions from one phase to another was not exceeded and thus avoiding situations in which nymphs changed stage during the experiment. The experiment was conducted at 25 ± 1° C, 70% ± 5% RH and 12:0 L:D and repeated for 17 times both for protonymphs (4 controls) and deutonymphs (5 controls), each with 4–5 controls, i.e. without predators. The number of replicates for the control treatment was determined on the basis of preliminary experiments in which no control mortality occurred in several replicates, or among active nor among chrysalis stages of the spider mite nymphs using the experimental procedure outlined above. Similarly no mortality occurred in the controls during the experiments.

### Data analyses

The assessment of preference was based on the formulae for Manly's preference index (Manly 1974):


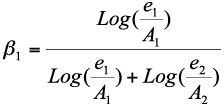


where *β*1 is the preference to prey type1, *e*1and *e*2 are the number of prey type1 and type2 (exp1: prey type1 is western flower thrips and prey type 2 is spider mites; exp2: prey type1 is active spider mite nymphs and prey type 2 is chrysalis spider mite nymphs) remaining after the experiment, Al and A2 are the number of prey type1 and type 2 presented to the predator. If the preference index is close to 1, the predator prefers prey type1, and if close to 0 the prey type2 is preferred. An index value close to 0.5 indicates no preference, i.e. predation is random (Cock 1978; Sherratt and Harvey 1993).

The number of consumed thrips larvae and spider mite protonymphs or deutonymphs as well as the number of consumed active and chrysalis stages of spider mite nymphs was compared by paired t-test. The prey preference indices were compared with nonparametric Mann-Whitney test. The mean numbers of consumed thrips and spider mites were transformed by square root before being subjected to data analyses. A significant level of α = 0.05 was used in all analyses.

## Results

### Prey preference between thrips and spider mites

*Amblyseius swirskii* clearly preferred western flower thrips first instar larvae both to spider mite protonymphs (t = 3.867, P = 0.001, df = 23) and deutonymphs (t = 5.820, P = 0.000, df = 20) with about twice as many thrips as spider mites being consumed at the end of the experiment (Table 1). Thus the preference indices for thrips (*β* (± SE): 0.69 ± 0.04 for the combination of thrips and spider mite protonymphs; *β* (± SE): 0.74 ± 0.04 for the combination of thrips and deutonymphs) differed significantly from those for spider mites (Mann-Whitney U value = 58, Z = 4.749, P = 0.000 for the former, and Mann-Whitney U value = 20, Z = -5.06, p = 0.000, respectively).

### Prey preference of A. swirskii between active and chrysalis stages of spider mite nymphs

*Amblyseius swirskii* did not exhibit a preference between the active and chrysalis stage of spider mite protonymphs (df = 16, t = -0.756 P = 0.460) or deutonymphs (df = 16, t = -1.282, P = 0.219) in spite of a slightly higher predation on the chrysalis stages (Table 2). Consequently, the preference indices for the active stage (*β* (± SE): 0.47 ± 0.05 for protonymphs; *β*(± SE): 0.41 ± 0.08 for deutonymphs) did not differ significantly from those for chrysalis stage (Mann-Whitney U value = 24.000, Z = -0.722, p = 0.470 for the former and Mann-Whitney U value = 98.5, Z = -1.598, p = 0.110, respectively).

**Table 1.**
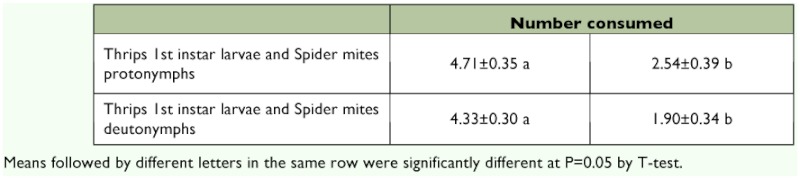
Mean number (±s.e.) of thrips first instar larvae and spider mite nymphs consumed in 24 h by adult female *Amblyseius swirskii*

*Amblyseius swirskii* had a higher predation on the active stages of spider mite protonymphs compared to the similar stage of deutonymphs (df = 16, t = 2.480, P = 0.025) and although the same was not the case for the chrysalis stages (df = 16, t = 1.367, P = 0.190), this difference was reflected in a difference in total consumption of the two nymphal instars of the spider mite (df = 16, t = 2.277, P = 0.037) (Table 2).

## Discussion

Our results confirmed that *A. swirskii* is capable of preying on spider mite nymphs with a consumption of 4–6 nymphs within a 12 h period. This is similar to results obtained by El-Laithy and Fouly (1992), who found that this predatory mite daily consumed 10.9 nymphs on mulberry leaves under conditions of 26° C and 70% RH comparable to those used here. It is, however, a little less than the daily consumption of spider mites (15 nymphs/day) by *A. swirskii* observed by Momen and El-Saway (1993), which presumably is a reflection of a higher temperature (27° C) and a longer starvation period (2 days) used by the latter authors.

*Amblyseius swirskii* consumed the same amount of the various types of spider mite nymphs except of the active deutonymphs of which significantly fewer were consumed. The latter is presumably in part a reflection of deutonymphs being larger and more active and thus more difficult to conquer and in part a reflection of a more pronounced congregating and web producing habit of deutonymphs compared to protonymphs (X. Xu personal observations) thereby hampering the movements of *A. swirskii.* The latter is in accordance with observations by van Houten et al. (2007a) that *Amblyseius swirskii* was hardly found in the webbing of *T. urticae.*

*Amblyseius swirskii* did not show any preference between the active and chrysalis stages of spider mite protonymphs or deutonymphs. The preferences determined in this study between first instar thrips and spider mite populations of predominately active proto- or deutonymphs can therefore be considered to also express the preference in cases of spider mite populations consisting of predominately chrysalis stages or with the two stages in more or less equal proportions.

Compared with either protonymphs or deutonymphs of spider mites, first instar thrips larvae were clearly preferred by *A. swirskii* with about twice as many thrips being consumed. However, the fact that the predatory mite may spend up to 25 % of its predation activities on spider mites when both pests occur simultaneously indicate that the predation pressure on thrips may be reduced perhaps leading to difficulties in obtaining satisfactory control of this pest. Additional studies are, however, needed to further elucidate the complex mechanisms between A *swirskii* and the number of its prey species (e.g. spider mites, thrips, and whiteflies) coexisting in many crops. In such multi-species environments several interactions besides direct predation, for instance apparent competition or apparent mutualism, may occur (Holt 1977) and influence the outcome of biocontrol. It has, for example, been shown that *A. swirskii* was able to reduce whitefly densities dramatically in situations where thrips were present compared to situations with absence of thrips resulting in an up to 15-fold increase in predator densities (Messelink et al. 2008). The authors attributed this to a positive influence of the mixed whitefly-thrips diet on the predator. Whether *A. swirskii* could also benefit from a mixed thrips-spider mite diet and whether this in longer-lasting biocontrol processes could compensate for the rather low daily predation on spider mites remains to be seen.

**Table 2.**
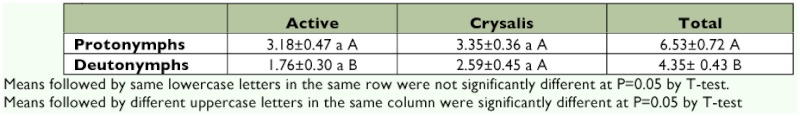
Mean number (± s.e.) of active and quiescent spider mite protonymphs and deutonymphs consumed in 12 h by adult female *Amblyseius swirskii*

Another aspect that relates to the outcome of biocontrol of thrips and spider mites with *A. swirskii* is the fact that the western flower thrips is a facultative predator of spider mite eggs (Trichilo and Leigh 1986). Although spider mites may avoid thrips-infested plant parts that would result in reducing this predation, and competition as well (Pallini et al. 1998), coexisting western flower thrips and spider mite populations may result in suppression of the spider mites (Messelink and Janssen 2008). A further complicating matter, however, is that the western flower thrips can also prey on eggs of predatory mites as has been documented for *Iphiseius degenerans* (Faraji et al. 2001, 2002), *Phytoseiulus persimilis* (Janssen et al. 1998) and *Amblyseius cucumeris* (Xu 2004). A similar predation on eggs of *A. swirskii* has not yet been examined but seems likely.

The present study was an initial and basic laboratory study undertaken to quickly examine the preference of *A. swirskii* under the most simplistic conditions. However, many factors such as plant architecture or plant traits (Krips et al. 1999; Pratt et al. 2002) may, of course, affect the searching and foraging behaviour of the predator and caution must consequently always be taken when results from such small scale laboratory experiments are used to predict interaction outcomes under greenhouse conditions.

Additional three-dimensional studies, on a small scale as well as long-term studies under more realistic large-scale conditions, on the biocontrol efficiency of *A. swirskii* against mixed infestations of thrips and spider mites will be needed for an evaluation of the practical consequences of the predator's preference for thrips and of the influence of the mixed diet and interactions between the pests and between the pests and *A. swirskii.*

## References

[bibr01] Argov Y, Domeratzky S, Gerson U, Steinberg S, Palevsky E (2007). Augmentation and conservation of indigenous generalist acarine predators for the control of citrus rust mite, *Phyllocoptruta oleivora,* in Israel.. *IOBC/wprs Bulletin*.

[bibr02] Bosco L, Giacometto E, Tavella L (2008). Colonization and predation of thrips (Thysanoptera: Thripidae) by *Orius* spp. (Heteroptera: Anthocoridae) in sweet pepper greenhouses in Northwest Italy.. *Biological Control*.

[bibr03] Brødsgaard HF, Enkegaard A, Pandalai SG (1997). Interactions among polyphagous anthocorid bugs used for thrips control and other beneficials in multi-species biological pest management systems.. *Recent Research Development in Entomology*.

[bibr04] CAB International (2007). *Animal Health and Production Compendium.*.

[bibr05] Calvo J, Fernandez P, Bolckmans K, Belda JE (2006). *Amblyseius swirskii* (Acari: Phytoseiidae) as a biological control agent of the tobacco whitefly *Bemisia tabaci* (Horn. : Aleyrodidae) in protected sweet pepper crops in Southern Spain.. *IOBC/wprs Bulletin*.

[bibr06] Cock MJW (1978). The assessment of preference.. *Journal of Animal Ecology*.

[bibr07] El-Laithy AYM, Fouly AH (1992). Life table parameters of the two phytoseiid predators *Amblyseius scutalis* (Athias-Henriot) and *A. swirskii* A.-H. (Acari, Phytoseiidae) in Egypt.. *Journal of Applied Entomology*.

[bibr08] El-Laithy AY (1998). Laboratory studies on growth parameters of three predatory mites associated with eriophyid mites in olive nurseries.. *Zeitschrift für Pflanzenkrankheiten und Pflanzenschutz*.

[bibr09] Escudero LA, Ferragut F (2005). Life-history of predatory mites *Neoseiulus californicus* and *Phytoseiulus per similis* (Acari: Phytoseiidae) on four spider mite species as prey, with special reference to *Tetranychus evansi* (Acari: Tetranychidae).. *Biological Control*.

[bibr10] Faraji F, Janssen A, Sabelis MW (2001). Predatory mites avoid ovipositing near counterattacking prey.. *Experimental and Applied Acarology*.

[bibr11] Faraji F, Janssen A, Sabelis MW (2002). Oviposition patterns in a predatory mite reduce the risk of egg predation caused by prey.. *Ecological Entomology*.

[bibr12] Fejt R, Jarosík V (2000). Assessment of interactions between the predatory bug *Orius insidiosus* and the predatory mite *Phytoseiulus persimilis* in biological control on greenhouse cucumber.. *Plant Protection Science*.

[bibr13] Helle W, Sabelis MW (1985). *Spider mites.*.

[bibr14] Holt RD (1977). Predation, apparent competition, and structure of prey communities.. *Theoretical Population Biology*.

[bibr15] Hoogerbrugge H, Calvo J, van Houten Y.M, Belda JE, Bolckmans K (2005). Biological Control of the Tobacco Whitefly *Bemisia tabaci* with the Predatory Mite *Amblyseius swirskii* in Sweet Pepper Crops.. *IOBC/wprs Bulletin*.

[bibr16] Hoogerbrugge H, Boer R, Messelink G (2009). Properties — combating which infestations.. http://www.allaboutswirskii.com/Combatingwhich-infestations.11661.0.html.

[bibr17] Jacobson R.J, Croft P, Fenlon J (2001). Suppressing Establishment of *Frankliniella occidentalis* Pergande (Thysanoptera: Thripidae) in Cucumber Crops by Prophylactic Release of *Amblyseius cucumeris* Oudemans (Acarina: Phytoseiidae).. *Biocontrol Science and Technology*.

[bibr18] Janssen A, Pallini A, Venzon M, Sabelis MW (1998). Review: Behavior and indirect interactions in food webs of plant-inhabiting arthropods.. *Experimental and Applied Acarology*.

[bibr19] Jannsen A, Faraji F, van der Hammen T, Magalhães S, Sabelis MW (2002). Interspecific infanticide deters predators.. *Ecology Letters*.

[bibr20] Krips OE, Kleijn PW, Willems PEL, Gols GJZ, Dicke M (1999). Leaf hairs influence searching efficiency and predation rate of the predatory mite *Phytoseiulus per similis* (Acari: Phytoseiidae).. *Experimental and Applied Acarology*.

[bibr21] Lewis T (1997). *Thrips as Crop Pests.*.

[bibr22] Manly BFJ (1974). A model for certain types of selection experiments.. *Biometrics*.

[bibr23] Messelink G, van Steenpaal S, van Wensveen W (2005). *Typhlodromips swirskii* (AthiasHenriot) (Acari: Phytoseiidae): a new predator for thrips control in greenhouse cucumber.. *IOBC/wprs Bulletin*.

[bibr24] Messelink G.J, van Steenpaal SEF, Ramakers MJ (2006). Evaluation of phytoseiid predators for control of western flower thrips on greenhouse cucumber.. *Biocontrol*.

[bibr25] Messelink G, Janssen A (2008). Do whiteflies help controlling thrips?. *IOBC/wprs Bulletin*.

[bibr26] Messelink G, van Maanen R, van Steenpaal SEF, Janssen A (2008). Biological control of thrips and whiteflies by a shared predator: two pests are better than one.. *Biological Control*.

[bibr27] Momen FM, El-Saway SA (1993). Biology and Feeding-Behavior of the Predatory Mite, *Amblyseius-Swirskii* (Acari, Phytoseiidae).. *Acarologia*.

[bibr28] Nomikou M., Janssen A, Schraag R, Sabelis MW (2001). Phytoseiid predators as potential biological control agents for *Bemisia tabaci.*. *Experimental and Applied Acarology*.

[bibr29] Nomikou M, Janssen A, Schraag R, Sabelis MW (2002). Phytoseiid predators suppress populations of *Bemisia tabaci* on cucumber plants with alternative food.. *Experimental and Applied Acarology*.

[bibr30] Nomikou M, Janssen A, Sabelis MW (2003). Phytoseiid predators of whiteflies feed and reproduce on non-prey food sources.. *Experimental and Applied Acarology*.

[bibr31] Pallini A, Janssen A, Sabelis MW (1998). Predators induce interspecific herbivore competition for food in refuge space.. *Ecology Letters*.

[bibr32] Parrella MP, Murphy B (1996). Western flower thrips: identification, biology and research on the development of control strategies.. *IOBC/wprs Bulletin*.

[bibr33] Pratt PD, Rosetta R, Croft BA (2002). Plantrelated Factors Influence the Effectiveness of *Neoseiulus fallacies* (Acari: Phytoseiidae), a Biological Control Agent of Spider Mites on Landscape Ornamental Plants.. *J. Econ. Entomol.*.

[bibr34] Ragusa S, Swirski E (1975). Feeding habits, development and oviposition of the predacious mite *Amblyseius swirskii* AthiasHenriot (Acarina: Phytoseiidae) on pollen of various weeds.. *Israel Journal of Entomology*.

[bibr35] Sengonca C, Zegula T, Blaeser P (2004). The suitability of twelve different predatory mite species for the biological control of *Frankliniella occidentalis* (Pergande) (Thysanoptera: Thripidae).. *Zeitschrift fur Pflanzenkrankheiten und Pflanzenschutz*.

[bibr36] Sherratt TS, Harvey I (1993). Frequencydependent food selection by arthropods: a review.. *Biological Journal of the Linnean Society*.

[bibr37] Swirski E, Amitai S, Dorzia N (1967). Laboratory studies on the feeding, development and reproduction of the predacious mites *Amblyseius rubini* Swirski and Amitai and *Amblyseius swirskii* Athias (Acarina: Phytoseiidae) on various kinds of food substances.. *Israel Journal of Agricultural Research*.

[bibr38] Tal C, Coll M, Weibtraub P (2007). Biological control of *Polyphagotarsonemus latus* by the predaceous mite *Amblyseius swirskii.*. *IOBC/wprs Bulletin*.

[bibr39] Tommasini MG, Maini S (2001). Thrips control on protected sweet pepper crops: enhancement by means of *Orius laevigatus* releases.. Thrips and Tospoviruses: Proceedings of the 7^th^ International Symposium on Thysanoptera.

[bibr40] Trichilo PJ, Leigh TF (1986). Predation on spider mite eggs by the western flower thrips, *Frankliniella occidentalis* (Thysanoptera: Thripidae), an opportunist in a cotton agroecosystem.. *Environmental Entomology*.

[bibr41] Trottin-Caudal Y, Leyre J-M, Baffert V, Fournier C, Chabriere C (2008). Experimental studies on *Typhlodromips* (*Amblyseius*) *swirskii* in greenhouse cucumber.. *IOBC/wprs Bulletin*.

[bibr42] van der Linden A (2004). *Amblyseius andersoni* Chant (Acari: Phytoseiidae), a successful predatory mite on Rosa spp.. *Communications in Agricultural & Applied Biological Sciences*.

[bibr43] van Houten YM, Ostilie ML, Hoogerbrugge H, Bolckmans K (2005). Biological control of western flower thrips on sweet pepper using the predatory mites *Amblyseius cucumeris,*
*Iphiseius degenerans, A. andersoni* and *A. swirskii.*. *IOBC/wprs Bulletin*.

[bibr44] van Houten YM, Hoogerbrugge H, Bolckmans KJF (2007a). Spider mite control by four phytoseiid species with different degrees of polyphagy.. *IOBC/wprs Bulletin*.

[bibr45] van Houten YM, Hoogerbrugge H, Bolckmans KJF (2007b). The influence of *Amblyseius swirskii* on biological control of twospotted spider mites with the specialist predator *Phytoseiulus persimilis* (Acari: Phytoseiidae).. *IOBC/wprs Bulletin*.

[bibr46] van Maanen R, Janssen A (2008). Prey preference of the generalist predator *Amblyseius swirskii.*. *IOBC/wprs Bulletin*.

[bibr47] Wilson LJ, Baure LR, Walter GH (1996). Phytophagous thrips are facultative predators of two spotted spider mites (Acari: Tetranychidae) on cotton in Australia.. *Bulletin of Entomological Research*.

[bibr48] Wimmer D, Hoffmann D, Schausberger P (2008). Prey suitability of western flower thrips, *Frankliniella occidentalis,* and onion thrips, *Thrips tabaci,* for the predatory mite *Amblyseius swirskii.*. *Biocontrol Science and Technology*.

[bibr49] Xu XN (2004). Combined releases of predators for biological control of spider mites *Tetranychus urticae* Koch and western flower thrips *Frankliniella occidentalis* (Pergande)..

[bibr50] El-Tawab AY, El-Keifl AH, Metwally AM (1982). Effect of temperature and photoperiod on the development, fecundity and longevity of *Amblyseius swirskii* Ath-Henr (Acari: Phytoseiidae).. *Anzeiger für Schädlingskunde, Pflanzenschutz, Umweltschutz*.

[bibr51] Zhang Z.-Q (2003). *Mites of Greenhouses, Identification, Biology and Control.*.

